# Crowd-counting technology within the Smart City context: understanding, trust, and acceptance

**DOI:** 10.3389/fpsyg.2024.1423837

**Published:** 2024-11-18

**Authors:** Theresa Waclawek, Angela Fiedler, Melissa Schütz, Astrid Schütz

**Affiliations:** Department of Psychology, University of Bamberg, Bamberg, Germany

**Keywords:** Smart City, urban overcrowding, crowd-counting technology, anonymization, trust, explanation, understanding, acceptance

## Abstract

In city centers worldwide, including the UNESCO World Heritage Site of Bamberg’s old town in Germany, alleviating pedestrian overcrowding is a pressing concern. Leveraging crowd-counting technologies with real-time data collection offers promising solutions, yet poses challenges regarding data privacy and informed consent. This preregistered study examines public response to a Smart City Bamberg project aimed at addressing pedestrian congestion through crowd-counting methods. We investigate informed consent by looking at understanding and acceptance of the project, as well as influencing factors, such as effectiveness of project explanation and trust. Through a three-stage study comprising exploratory interviews, a field study, and an online study, we reveal that the focus of project explanations significantly impacts understanding: Functional explanations, emphasizing project purpose, enhance comprehension compared to mechanistic explanations detailing project components. Additionally, project trust positively correlates with acceptance. Notably, understanding impacts acceptance through increased project trust. These findings underscore the importance of fostering understanding to garner public acceptance of crowd-counting projects. It is important, especially in the case of projects which aim to improve quality of life while also prioritizing robust data protection, that decisions regarding informed consent are grounded in comprehension rather than on preconceived biases against data sharing. Efforts should prioritize effective explanations to bolster project trust and consequently, promote acceptance.

## 1 Introduction

The old town of Bamberg, Germany is a UNESCO world heritage site (UNESCO World Heritage Centre, 2024), and attracts large numbers of tourists, with almost 8 million day tourists annually (“Bamberg - Zahlen Und Fakten,” 2023). This can lead to issues of pedestrian overcrowding, which can prove stressful (Rodriguez-Valencia et al., 2022; Zhang et al., 2023). Technological advancements are useful to manage and avoid overcrowding (Sharma et al., 2018) and enhance well-being in busy pedestrian areas (Mouratidis, 2021). However, these methods often involve data collection, which can entail privacy risks (Singh et al., 2021), and therefore require public acceptance. Within the context of Smart City Bamberg, we conducted three studies to better understand public acceptance of a project using crowd-counting methods to provide data-driven visitor guidance. This involves passive data collection using Wi-Fi sensors (Ackermann et al., 2023). Though the stored data are anonymized, the sensors detect MAC addresses, which are considered personal data (Ackermann et al., 2023). According to Recital 32 of the GDPR, data collection involving personal data necessitates informed consent. Therefore, we aim to explore issues surrounding informed consent. Specifically, we address the following research question: which conditions promote both understanding and acceptance of this data collection? Currently, there is a dearth of research on these issues relating to crowd-counting methods. We conducted exploratory interviews, a field study and an online study; our hypotheses are informed by existing literature and our own exploratory analyses. This project was preregistered with the Open Science Framework (doi: 10.17605/OSF.IO/ATNYB; which also contains study materials).

It is important that data collection in crowd-counting projects is explained in an understandable manner to enable informed consent. Therefore, we investigated how different explanations impact understanding, both subjectively (perceived comprehension) and objectively (performance in understanding tests). Prior research indicates that varying aspects of explanations impacts subjective understanding, such as their interactivity (Cheng et al., 2019), accessibility of additional information (Sloman and Rabb, 2016), and media format (Wen et al., 2023).

We manipulated explanations based on the established distinction between functional and mechanistic explanations (Lombrozo and Wilkenfeld, 2019). Functional explanations appeal to goals and purposes, while mechanistic explanations appeal to parts and processes (Lombrozo and Wilkenfeld, 2019). Research suggests a preference in participants for functional explanations (Joo et al., 2022; McCarthy and Keil, 2023). It is not clear why this preference exists. One possibility is that functional explanations elicit a greater sense of understanding. In a recent study, participants rated their understanding lower when there was a mechanistic compared to a functional framing (Zemla and Corral, 2023). However, it is not yet known how functional or mechanistic explanations impact subjective and objective understanding. This is especially relevant for Smart City projects because explanations are a major tool for promoting informed consent. Therefore, we hypothesized that:

*H1 a) Functional explanations elicit greater subjective understanding.*

Additionally, we examined the relationship between type of explanation and objective understanding. Based on our exploratory analyses from the field study, we predicted that:

*H1 b) There is no relationship between type of explanation and objective understanding.*

Initially, we had also hypothesized that explanation type impacts acceptance, such that functional explanations elicit higher acceptance. Previous research has suggested that communicating the implications of data sharing versus the method of data handling affects data sharing decisions (Xiong et al., 2020). However, we did not find evidence for this in the field study, and therefore did not hypothesize this result for the online study.

In seeking informed consent for data collection, fostering understanding and acceptance should occur in tandem. Therefore, we also aimed to uncover factors contributing to project acceptance. Prior research underscores the role of trust in acceptance of technology (e.g., Choung et al., 2023; Dhagarra et al., 2020; Keusch et al., 2019).

The concept of trust, however, is contextualized: For example, one can differentiate between trust in humans versus trust in technology (e.g., Julsrud and Krogstad, 2020; Kessler et al., 2017; Mcknight et al., 2011). For this reason, we first, through our exploratory interviews, asked interviewees about important factors when deciding to share location data, and then developed a measure of trust in relation to the issues raised. This project-related trust measure was then employed in the subsequent field and online studies. By determining what is relevant to trust in crowd-counting technologies, we were able to explore the established relationship between trust and acceptance in a novel context, and hypothesized that:

*H2) Project trust is positively related to acceptance of the project.*

Acknowledging this established relationship between trust and acceptance, it is imperative to investigate the determinants of project trust. Exploratory analyses during our field study led us to hypothesize that understanding bolsters trust, and this in turn fosters acceptance—and that this would be true for subjective and objective understanding. The relationship between understanding and acceptance is important in informed consent, however, it is not yet known how understanding impacts acceptance in the context of crowd-counting technologies, nor are mechanisms yet understood. Therefore, we predicted that:

*H3 a) Subjective understanding of the crowd counting method is positively associated with public acceptance of these methods, and this relationship is mediated by higher levels of trust in the project.*

*H3 b) Objective understanding of the crowd counting method is positively associated with public acceptance of these methods, and this relationship is mediated by higher levels of trust in the project.*

## 2 Study 1: exploratory interviews

### 2.1 Methods

We chose a three-stage approach to investigate our hypotheses. Initially, we conducted exploratory interviews to determine factors relevant to data sharing. The sample for study 1 consisted of randomly selected people on the street in Bamberg who agreed to participate (*n* = 58). All interviewers followed an interview guide that focused primarily on questions relating to participants’ understanding of and concerns related to data sharing. The interviews were qualitatively analyzed using MAXQDA (VERBI Software, 2022), first independently by three of the co-authors, and then the emergent themes were discussed and agreed upon.

### 2.2 Results and discussion

The most prominent themes to emerge related to the storage of personal data, if there is access to the data by third parties, if the data are used for commercial means, if it is likely that the data collection could be harmful to some, and if the data collection is likely to be useful to some. These themes were then used as a basis for the development of the scale for project trust.

## 3 Study 2: field study

### 3.1 Methods

Our field study was conducted on the streets of Bamberg, and consisted of randomly selecting people (*n* = 90) who could take part in a lottery for vouchers (see Table 1).

**Table 1:** Demographic information.

Study	Gender	Age	Number of students
Exploratory interviews	*n* = 32 male	*n* = 2 age bracket <20	*n* = 21
		*n* = 23 age bracket 20–30	
	*n* = 25 female	*n* = 7 age bracket 31–40	
		*n* = 6 age bracket 41–50	
	*n* = 1 other/N.A.	*n* = 10 age bracket 51–60	
		*n* = 10 age bracket >60	
Field study (*n* = 90)	*n* = 30 male	*M* = 38.33	*n* = 41
	*n* = 59 female	*SD* = 19.13	
	*n* = 1 other/N.A.		
Online study (*n* = 197)	*n* = 42 male	*M* = 24.91	*n* = 161
	*n* = 153 female	*SD* = 7.54	
	*n* = 2 other/N.A.		

The field study employed an experimental manipulation in which participants were presented with one of two explanations. Each explanation consisted of four pictures with short verbal explanations. The mechanistic explanation focused on the parts and processes of the project and described how the sensors collect and store the data. The functional explanation focused on the purpose of the data collection and described the goal of offering services to improve pedestrian traffic. Participants were randomly assigned to conditions.

Subjective understanding was assessed using the question “Do you think you understand what the sensors do and why they do it?” It was rated on a four-point scale from “No” to “Yes, I understand well.”

Objective understanding was measured by asking participants to describe in open-answer format what they understood from the explanation. Answers were scored for correct information.

Project trust was assessed using the scale developed from the exploratory interviews. Five items were evaluated on a scale ranging from 1 (complete distrust) to 11 (complete trust). An example question is: “If your Wi-Fi is switched on near a sensor as described above, how much do you trust that no personal data is stored?” The internal consistency of the scale was good, with a Cronbach’s alpha of 0.87 (95% CI [0.83, 0.91]).

Acceptance was assessed with one item asking, “Would you be willing to share your location data (as explained earlier) with this project by activating the Wi-Fi function of your mobile device near a sensor?” Participants could answer with no, maybe or yes.

All analyses for both the field and the online study were performed using R statistical software (version 4.3.1) using packages including: psych (Revelle, 2024), MASS (Venables and Ripley, 2002), and mediation (Tingley et al., 2014).

### 3.2 Results

The result from the ordinal regression investigating whether functional explanations yield better subjective understanding compared to mechanistic explanations was not significant. Nonetheless, the odds ratio for moving one category up in subjective understanding on the four-point Likert scale was positive at 1.95 [95% CI: 0.83, 4.60], suggesting that functional explanations tend to lead to greater subjective understanding. The results also indicated a non-significant effect of explanation condition on acceptance, with an odds ratio for moving one category up in acceptance on the four-point Likert scale of 1.2 [95% CI: 0.53, 2.74]. Lastly, they indicated that more project trust is associated with higher acceptance, with an odds ratio of 1.59 [95% CI: 1.30, 1.96].

### 3.3 Discussion

This field study allowed for initial testing of our hypotheses, as well as exploratory analyses for further hypothesis development, and for methodological refinement. We decided to further test the hypothesis regarding the relationship between trust and acceptance in our online study. Additionally, visual inspection of the relationship between explanation condition and subjective understanding indicated an effect of condition on understanding, and so we decided to further test this. Due to a lack of findings of explanation condition on acceptance, we did not test this again. We used exploratory analyses to develop additional hypotheses, which indicated a possible mediation between understanding (both subjective and objective) and acceptance through a positive relationship with project trust. We checked for possible moderating roles of age, gender, as well as self-rated importance of understanding, but only found low correlations and did not include these possible control variables in our online study. Furthermore, our objective understanding measure could allow for those who are more cooperative to give longer answers, and therefore score more highly. Therefore, we decided to change the answer format from open answer to multiple choice. We tested all of this with a larger sample and under anonymous conditions.

## 4 Study 3: online study

### 4.1 Methods

An *a priori* power analysis with the shiny app “Monte Carlo Power Analysis for Indirect Effects” (Schoemann et al., 2017) and values from our field study provided a minimum sample size of *n* = 194 participants for a power of 80% (95% CI [75, 84%]). With this sample size, the minimum detectable effect size for the indirect path was *r*(*ab*) = 0.07.

The final sample included *n* = 197 people (see Table 1). We checked all scales for outliers (3 *SD* above or below the mean), but did not have exclusions. Participants could enter a lottery for vouchers, and student participants could receive course credits.

The experimental variation and the measurement of variables were the same as in the field study, with a few adjustments. We used the same scale for project trust, and in this study obtained a Cronbach’s alpha of 0.81 (95% CI [0.76, 0.85]). Due to the options available on the online platform we were able to make use of a pseudo-continuous scale for all outcome variables used in this analysis (a sliding scale with 100 values), which could capture variation in constructs such as feeling of understanding with greater sensitivity. Participants saw only the anchor statements and a sliding scale without numbers. We also adapted the objective understanding question into multiple choice to account for the issue raised above. Participants responded to five multiple-choice questions and scoring accounted for explanation condition. All participants answered the same questions, but the correct answers varied depending on the explanation condition (e.g., in some cases, “information not provided by the explanation” was the correct response for one condition, while the other group had access to this information, and in some cases the same answer was correct for both conditions). A higher score indicated greater objective understanding. The internal consistency of the scale was deemed acceptable, with a Cronbach’s alpha of 0.71 (95% CI [0.64, 0.77]). For reporting, all outcomes were normalized to a 0–10 scale for comparability (Table 2).

**Table 2:** Means and standard deviations of understanding, trust, and acceptance, trust by study and explanation condition.

Measure	Field study		Online study	
	Functional (*n* = 41)	Mechanistic (*n* = 48)	Functional (*n* = 94)	Mechanistic (*n* = 103)
Subjective understanding	8.86 (1.77)	7.78 (3.02)	7.88 (2.07)	7.07 (2.23)
Objective understanding	N.A.	N.A.	7.74 (1.55)	6.95 (1.30)
Trust	5.82 (2.43)	5.57 (2.27)	5.22 (2.11)	5.18 (2.09)
Acceptance	6.75 (3.05)	6.28 (3.27)	5.64 (3.18)	4.92 (2.86)

All scales have been normalized to the range 0–10. Values in brackets are standard deviations.

### 4.2 Results

A Welch’s two-sample *t*-test found greater subjective understanding in the functional explanation group with a small effect size [*d* = 0.38, *t*(195) = −2.64, *p* < 0.01] and greater objective understanding with a medium effect size [*d* = 0.52, *t*(180) = −3.59, *p* < 0.001]. The correlation between subjective and objective understanding was small [*r*(195) = 0.25, *p* < 0.001].

A linear regression analysis supported the hypothesis that project trust is associated with acceptance [*B* = 0.5, *R*^2^ = 0.12, *F*(1, 190) = 25.99, *p* < 0.001].

**Figure 1:**
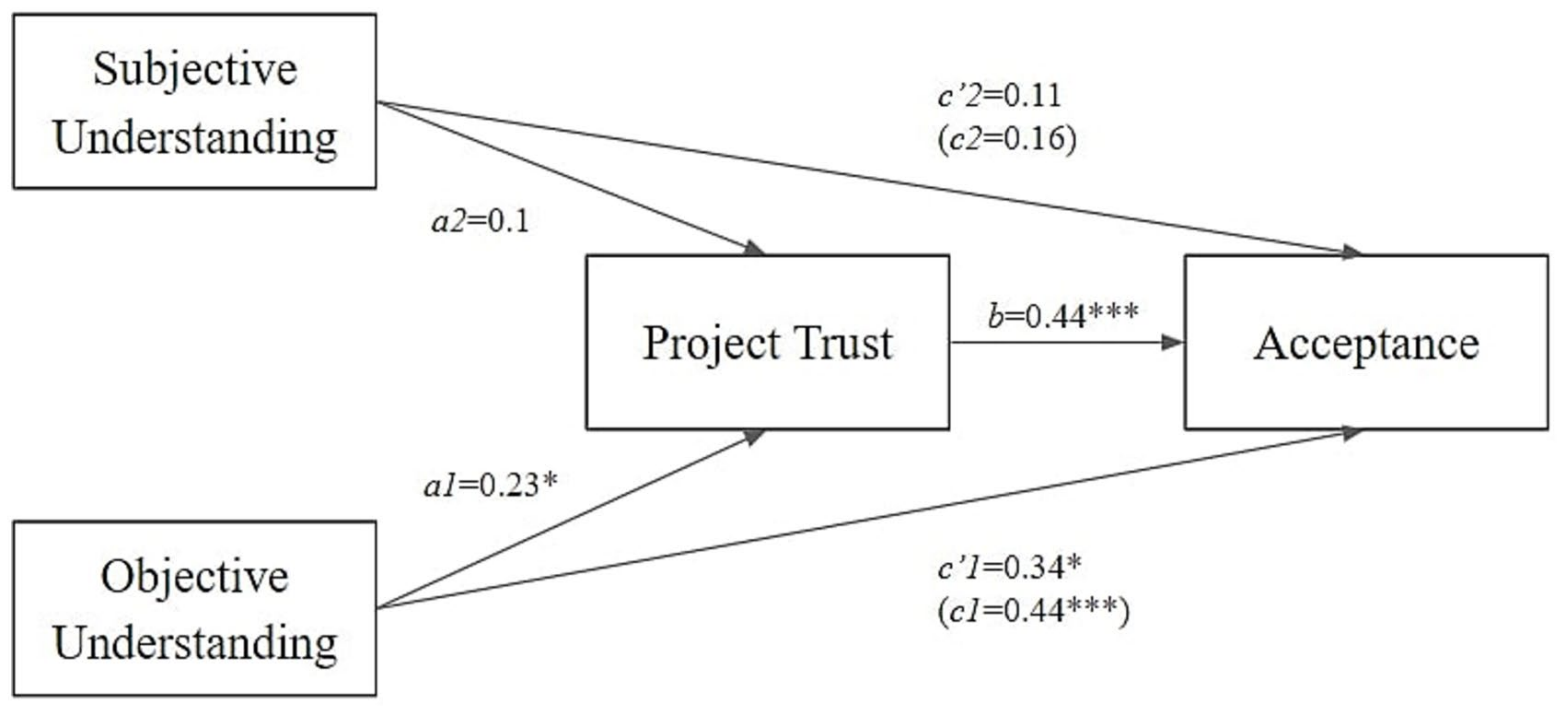
The mediation analysis of the relationship between understanding and acceptance, with project trust as the mediator. *p ≤ 0.05, **p ≤ 0.01, ***p ≤ 0.001.

We also found support for the link between understanding with trust and acceptance. Using the PROCESS macro (Hayes, 2022) we included both subjective and objective understanding as predictor variables and trust as the mediator variable. The total effect of objective understanding on acceptance through trust was significant (*c1* = 0.44, *p* = 0.005), the direct effect was significant (*c’1* = 0.34, *p* = 0.023), and the indirect effect through trust was significant (*ab1* = 0.10, 95% CI [0.01, 0.22]). Neither the total effect of subjective understanding on acceptance through trust (*c2* = 0.16, *p* = 0.113), the direct effect (*c’2* = 0.11, *p* = 0.236), nor the indirect effect (*ab2* = 0.05, 95% CI [−0.02, 0.12]) were significant (Figure 1; Table 3).

**Table 3:** Coefficients, standard errors, and confidence intervals of the models used in the mediation analysis of the relationship between understanding and acceptance, with project trust as the mediator.

Variable	Project trust			Acceptance		
	*B*	*SE*	95% CI	*B*	*SE*	95% CI
Subjective understanding	0.10	0.07	[−0.04, 0.24]	0.11	0.09	[−0.08, 0.3]
Objective understanding	0.23*	0.11	[0.02, 0.44]	0.34*	0.15	[0.05, 0.63]
Project trust				0.44***	0.1	[0.25, 0.64]
	*R*^2^ = 0.04			*R*^2^ = 0.157		
	*F*(2, 189) = 4.34, *p* = 0.01			*F*(3, 188) = 11.67, *p* < 0.001		

*p ≤ 0.05, **p ≤ 0.01, ***p ≤ 0.001.

Since we had found effects of explanation condition on both subjective and objective understanding, we ran an additional exploratory analysis to look at the relationship between subjective understanding and acceptance with objective understanding and then trust as sequential mediators. There was a total effect of *d* = 0.23 (*p* = 0.023) and an indirect effect from subjective understanding to acceptance through objective understanding of *ab* = 0.05 (95% CI [0.01, 0.11]) and then through first objective understanding and then through trust of *abc =* 0.02 (95% CI [0.001, 0.037]).

We conducted an additional exploratory mediation with explanation condition as the independent variable, and, following from the above exploratory analysis, we included the sequential mediating variables of subjective understanding, objective understanding, and trust, with acceptance as the outcome. The total effect of explanation condition equaled 0.75 (*p* = 0.089). Regarding indirect pathways, we found a possible mediation from explanation to acceptance through objective understanding (effect = 0.19, 95% CI [0.01, 0.45]), as well as through objective understanding and then trust (effect = 0.07, 95% CI [0.004, 0.187]), as well as through subjective understanding, objective understanding, and then through trust [effect = 0.01 (95% CI, [0.00, 0.03])]. The confidence intervals of all other pathways included 0.

### 4.3 Discussion

These results support our prediction that functional explanations result in better understanding, both subjectively and objectively. In the preregistered mediation, understanding did predict acceptance through project trust, but this was better explained through increases in objective understanding rather than subjective understanding. The exploratory analysis following these results suggest that subjective understanding may impact acceptance, but that this could be mediated by objective understanding as well as by trust. Exploratory results further suggest that this pathway may begin with explanation type, followed by better subjective understanding, objective understanding, higher project trust, and finally, increased acceptance.

## 5 Discussion

In this study, we investigated public response to the Smart City project aiming to improve pedestrian traffic using crowd counting technology. We shed light on the relationships between explanation, understanding, trust, and acceptance and discuss each of the main hypotheses below.

### 5.1 Functional explanations produce greater understanding than mechanistic explanations

We found that explanation type impacts understanding, with functional explanations yielding greater subjective and objective understanding. These findings align with previous research but also provide new evidence. That functional explanations result in greater understanding is consistent with the findings that there is a preference for functional over mechanistic explanations (Joo et al., 2022; McCarthy and Keil, 2023) and that participants report lower subjective understanding when asked about how an object works when this object was framed in a mechanistic context (Zemla and Corral, 2023). Our findings add to this by providing evidence that presenting participants with functional over mechanistic explanations results in both better subjective and objective understanding.

This provides valuable insight into how to explain crowd counting technologies to promote informed consent. Interestingly, it has been previously demonstrated that people prefer functional explanations to precede mechanistic explanations (McCarthy and Keil, 2023). Research suggests that perceived learning is linked to satisfaction with an explanation (Liquin and Lombrozo, 2022). Importantly, satisfaction has been related to further curiosity (Liquin and Lombrozo, 2022). Therefore, if functional explanations of crowd-management projects foster a greater feeling of understanding, which is associated with satisfaction, these explanations may also stimulate curiosity about important related questions also important to understanding, such as those related to mechanistic information. Therefore, from our findings, it may be beneficial to present crowd members first with functional explanations, and then with mechanistic information, as such an approach could enhance understanding and promote curiosity.

One caveat to consider in our experimental manipulation is the use of technical language included in our mechanistic explanation. The use of jargon, even when defined, can negatively impact ease of processing information (Shulman et al., 2020), as well as learning success (McDonnell et al., 2016). Conversely, the use of technical terms has also been found to increase satisfaction with an explanation (Hopkins et al., 2016), to improve perceived understanding (Rhodes et al., 2014) and was believed to provide explanatory content even when it did not (Liquin and Lombrozo, 2022). It is unclear, then, in which direction the inclusion of technical terms in our mechanistic explanation may have influenced subjective understanding, and this should be disentangled in future.

Understanding is important in and of itself, but it also relates to other important outcomes of crowd-management efforts, such as trust in and acceptance of data collection.

### 5.2 Project trust is associated with greater acceptance

As predicted, project trust demonstrated a significant association with project acceptance. This finding is consistent with a well-established body of literature (Choung et al., 2023; Dhagarra et al., 2020; Julsrud and Krogstad, 2020; Kelly et al., 2023; Keusch et al., 2019).

Beyond this, our study contributes additionally by investigating what is important for trust of data collection within a Smart City project. We developed a measure of project trust based on concerns expressed during our interview study which included themes such as data protection (i.e., no access by third parties, no storage of personal data, and no use of data for commercial means), as well as the general harmfulness or helpfulness of the data collection. Both the insights gained from the interviews and the observed association of this measure with acceptance elucidate which information may be important to highlight in the communication of crowd-management projects to encourage project trust and, consequently, acceptance.

This relationship between project trust and acceptance also fits into the larger framework of our study. As discussed previously, we can impact understanding through explanation. In the exploratory analyses following the field study, we observed a pattern indicating a potential mediating relationship wherein understanding affected acceptance through project trust, which we elaborate on below.

### 5.3 Understanding is associated with greater acceptance through higher project trust

How understanding of Smart City crowd-counting projects relates to acceptance, and what might underlie this relationship had not been previously explored, and so we contribute with the finding that objective understanding, but not subjective understanding, is positively associated with acceptance through increased project trust. Though both aspects of understanding were impacted by explanation type, it seems that actual rather than felt understanding can better explain differences in attitude and behavior.

There exists hesitancy among some individuals to participate in data collection within a Smart City context (Van Twist et al., 2023; Julsrud and Krogstad, 2020). While privacy and security concerns are frequently cited reasons for reluctance to engage in passive mobile data collection (Keusch et al., 2019; Revilla et al., 2019), effective communication of relevant information has been identified as a key component in fostering acceptance of beneficial Smart City technologies (Van Twist et al., 2023; Cabalquinto and Hutchins, 2020; Peng et al., 2017; Tang et al., 2021). In our study, the described crowd-counting project was designed to promote the well-being of residents and tourists in Bamberg, evident through its goals of reducing pedestrian overcrowding and ensuring protection of personal data through robust anonymization (Ackermann et al., 2023). Given the project’s emphasis on robust anonymization, it is important that individuals’ decisions regarding data sharing are based on a good understanding of the project rather than on predetermined biases against data sharing.

But how can we create the right conditions for this? From our first two hypotheses, we know that explanation type influences understanding, and that trust is associated with acceptance. With our third hypothesis we bridge understanding, trust, and acceptance, wherein understanding is positively associated with acceptance through higher project trust. This produces the actionable suggestion that crowd-counting projects can focus on choosing explanations that promote understanding, and that this increased understanding may increase trust and therefore acceptance. We found preliminary evidence for this in our exploratory analyses, in which there was a notable indirect pathway from explanation condition, through subjective and then objective understanding, and then through project trust to acceptance. Therefore, in the context of projects promoting socially desirable outcomes and prioritizing data protection, we contribute to knowledge regarding how to effectively inform stakeholders so as to promote informed consent.

### 5.4 Limitations and future research directions

A limitation of our study is the distribution of our samples. While the field study was conducted in Bamberg and thus sampled from the population of interest, almost half of the participants were students and there was an imbalance in gender representation, with approximately double the amount of people identifying as female compared to male. This skew was even more pronounced in the online study. Another limitation is that we did not investigate some possible moderators. While we did explore the roles of age, gender, and importance of understanding following our field study, and only observed negligible to low correlations, future research can look at people’s existing attitudes toward, and current understanding of technology.

Future research directions can include further manipulations of explanation to develop a more refined understanding of which explanations would be best in the context of Smart City or crowd-counting projects. As mentioned, it will be important to examine the use of technical language; these projects involve technical components, and we need a clearer picture regarding how best to communicate this understandably. Additionally, as such projects often involve multiple public and private contributors, an area for future research relates to how institutional representation impacts trust and acceptance. Academic sources are considered trustworthy institutions with which to share location data (Leder et al., 2022; Keusch et al., 2019). Therefore, future research could investigate the impact of other institutions on trust and acceptance of crowd-counting technologies. Also, the results are limited to this specific method of Wi-Fi data collection. It is still unclear whether other methods might lead to different results, as aspects such as familiarity with a certain technology could lead to greater trust, which has already been shown in the context of AI-technology (Horowitz et al., 2024).

## 6 Conclusion

In this study, we investigated public perception of a crowd-counting project aimed at reducing pedestrian overcrowding in the UNESCO world heritage site of the old town of Bamberg, Germany. We explored themes related to explanation, understanding, trust, and acceptance. Our findings shed light on factors that can enhance the informed consent of Smart City projects. First, we found that explanation significantly impacts understanding, suggesting that offering explanations highlighting the purpose of the project (the functional explanation) as initial messaging could be beneficial. Secondly, our study underscores the importance of trust in the acceptance of such projects. Trust emerges as an important factor in public acceptance, highlighting the need for strategies to build and maintain trust in Smart City initiatives. Lastly, promoting understanding of projects with socially desirable aims and a commitment to data protection may enhance trust and, consequently, acceptance of such projects. Overall, our findings provide valuable insights for the development and implementation of Smart City projects, emphasizing the significance of effective communication to ensure informed consent.

## Data Availability

The raw data supporting the conclusions of this article will be made available by the authors, without undue reservation.
