# Supplementary material for: Crowd-counting technology within the Smart City context: understanding, trust, and acceptance
Source: Front Psychol. 2024 Nov 18;15:1423837. doi: 10.3389/fpsyg.2024.1423837 (PMC11608974; doi:10.3389/fpsyg.2024.1423837)
Supplement: Supplementary file 1 [file Data_Sheet_1.docx]

**Supplementary Materials**

| Variable | | Objective Understanding | | | | Project Trust | | | | Acceptance | | |
| --- | --- | --- | --- | --- | --- | --- | --- | --- | --- | --- | --- | --- |
|  | *B* | | *SE* | 95% CI | *B* | | *SE* | 95% CI | *B* | | *SE* | 95% CI |
| Subjective Understanding | 0.15** | | 0.05 | 0.06, 0.24 | 0.10 | | 0.07 | -0.04, 0.24 | 0.11 | | 0.10 | -0.08, 0.10 |
| Objective Understanding |  | |  |  | 0.23* | | 0.11 | 0.01, 0.44 | 0.34* | | 0.15 | 0.05, 0.64 |
| Project Trust |  | |  |  |  | |  |  | 0.44*** | | 0.10 | 0.25, 0.64 |
| *R*² = 0.06  *F*(1, 190) =11.2, *p*= 0.001 | | | | *R*² = 0.04  *F*(2, 189) =4.34, *p*=0.01 | | | | *R*² = 0.16  *F*(3, 188) =11.67, *p*=0.000 | | |  |  |

**Supplementary Table 1:** Coefficients, standard errors and confidence intervals of the models used in the exploratory mediation analysis of the relationship between subjective understanding and acceptance, with objective understanding and project trust as sequential mediations. *Note:* **p* ≤ 0.05, ***p* ≤ 0.01, ****p* ≤ 0.001.

| Variable | Subjective Understanding | | | | Objective Understanding | | | Project Trust | | | Acceptance | | |  |
| --- | --- | --- | --- | --- | --- | --- | --- | --- | --- | --- | --- | --- | --- | --- |
|  | *B* | *SE* | 95% CI | *B* | | *SE* | 95% CI | *B* | *SE* | 95% CI | *B* | *SE* | 95% CI | |
| Explanation | 0.79* | 0.31 | 0.17, 1.41 | 0.62** | | 0.20 | 0.23, 1.01 | -0.18 | 0.31 | -0.80, 0.43 | 0.41 | 0.42 | -0.43, 1.25 | |
| Subjective Understanding |  |  |  | 0.13** | | 0.05 | 0.03, 0.22 | 0.11 | 0.07 | -0.03, 0.25 | 0.10 | 0.10 | -0.09, 0.29 | |
| Objective Understanding |  |  |  |  | |  |  | 0.25* | 0.11 | 0.03, 0.46 | 0.31* | 0.15 | 0.01, 0.61 | |
| Project Trust |  |  |  |  | |  |  |  |  |  | 0.45*** | 0.10 | 0.25, 0.65 | |
| *R*² = 0.03  *F*(1, 190) = 6.37, *p*=0.012 | | | | *R*² =  0.10  *F*(2, 189) = 10.7 , *p*= 0.000 | | | *R*² = 0.05.  *F*(3, 188) = 3.0 , *p*=0.032 | | | *R*² = .16  *F*(4, 187) = 8.98, *p*=0.000 | | |  |  |

**Supplementary Table 2:** Coefficients, standard errors and confidence intervals of the models used in the mediation analysis of the relationship between explanation type and acceptance, with subjective understanding, objective understanding, and project trust as sequential mediations. *Note:* **p* ≤ 0.05, ***p* ≤ 0.01, ****p* ≤ 0.001.
